# Trichloroethylene Exposure during Cardiac Valvuloseptal Morphogenesis Alters Cushion Formation and Cardiac Hemodynamics in the Avian Embryo

**DOI:** 10.1289/ehp.8781

**Published:** 2006-02-09

**Authors:** Victoria J. Drake, Stacy L. Koprowski, John Lough, Norman Hu, Susan M. Smith

**Affiliations:** 1 Department of Nutritional Sciences, University of Wisconsin–Madison, Madison, Wisconsin, USA; 2 Department of Cell Biology, Neurobiology, and Anatomy, Medical College of Wisconsin, Milwaukee, Wisconsin, USA; 3 Department of Pediatrics, University of Utah, Salt Lake City, Utah, USA; 4 Primary Children’s Medical Center, Salt Lake City, Utah, USA

**Keywords:** cardiac cushions, chick embryo, Doppler ultrasound, heart development, proliferation, trichloroethylene

## Abstract

It is controversial whether trichloroethylene (TCE) is a cardiac teratogen. We exposed chick embryos to 0, 0.4, 8, or 400 ppb TCE/egg during the period of cardiac valvuloseptal morphogenesis (2–3.3 days’ incubation). Embryo survival, valvuloseptal cellularity, and cardiac hemodynamics were evaluated at times thereafter. TCE at 8 and 400 ppb/egg reduced embryo survival to day 6.25 incubation by 40–50%. At day 4.25, increased proliferation and hypercellularity were observed within the atrioventricular and outflow tract primordia after 8 and 400 ppb TCE. Doppler ultrasound revealed that the dorsal aortic and atrioventricular blood flows were reduced by 23% and 30%, respectively, after exposure to 8 ppb TCE. Equimolar trichloroacetic acid (TCA) was more potent than TCE with respect to increasing mortality and causing valvuloseptal hypercellularity. These results independently confirm that TCE disrupts cardiac development of the chick embryo and identifies valvuloseptal development as a period of sensitivity. The hypercellular valvuloseptal profile is consistent with valvuloseptal heart defects associated with TCE exposure. This is the first report that TCA is a cardioteratogen for the chick and the first report that TCE exposure depresses cardiac function. Valvuloseptal hypercellularity may narrow the cardiac orifices, which reduces blood flow through the heart, thereby compromising cardiac output and contributing to increased mortality. The altered valvuloseptal formation and reduced hemodynamics seen here are consistent with such an outcome. Notably, these effects were observed at a TCE exposure (8 ppb) that is only slightly higher than the U.S. Environmental Protection Agency maximum containment level for drinking water (5 ppb).

Trichloroethylene (TCE; C_2_HCl_3_) is a chlorinated hydrocarbon used predominantly as an industrial degreasing agent and solvent (Armstrong and Green 2004; Waters et al. 1977; Wu and Schaum 2000). Human exposures are widespread, and TCE is a major contaminant in agricultural and urban soils and in groundwater (Wallace et al. 1987; Wu and Schaum 2000). The current U.S. Environmental Protection Agency (EPA) maximum contaminant level (MCL) for TCE in drinking water is 5 ppb (U.S. EPA 2004). TCE levels in contaminated water supplies have exceeded 230 ppb [Goldberg et al. 1990; Massachusetts Department of Public Health (MDPH) 1996]. Chlorination of water supplies generates several TCE metabolites, including chloral hydrate, trichloroacetic acid (TCA), and dichloroacetic acid (Miller and Uden 1983). These metabolites are also formed *in vivo* from TCE. Because most public water systems in the United States use chlorine for disinfection (U.S. EPA 1997), exposures to these metabolites can be significant.

There is lively debate whether maternal exposure to TCE and related compounds increases the risk for congenital heart defects (CHDs) in the offspring. Human epidemiologic studies are limited and inconclusive (Bove et al. 1995; Goldberg et al. 1990; MDPH 1996; Shaw et al. 1990, 1992; Yauck et al. 2004). The most common CHDs reported involve valvuloseptal structures and include ventricular and atrial septal defects and aortic and pulmonary valve stenosis (Goldberg et al. 1990; Yauck et al. 2004).

Results from animal studies are also contradictory. Some report no effect of TCE exposure (Dorfmueller et al. 1979; Fisher et al. 2001; Schwetz et al. 1975), whereas others report a higher incidence of CHDs, especially those of valvuloseptal origin (Dawson et al. 1990, 1993; Johnson et al. 1998a, 2003). Valvuloseptal deficits also occur in an avian embryo model of TCE exposure (Loeber et al. 1988). The mechanism(s) by which TCE would cause CHDs is under investigation. TCE may directly target valvuloseptal formation; in a chick atrioventricular canal (AVC) explant system, 250 ppm TCE significantly inhibited the epithelial–mesenchymal cell transformation that underlies chamber septation and valve formation (Boyer et al. 2000). TCE exposure also alters the expression of several genes critical for heart development (Boyer et al. 2000; Collier et al. 2003; Ou et al. 2003; Selmin et al. 2005).

The heart develops as a linear tube, composed of an inner endocardium and an outer myocardium that is separated by an extracellular matrix. Shortly thereafter, the matrix at two sites within the heart tube, the outflow tract (OFT) and AVC, becomes cellularized to form “cardiac cushions” (Person et al. 2005). During this process, endocardial cells respond to myocardial signals and migrate from the endocardium into the extracellular matrix, where they adopt a mesenchymal phenotype in a process called epithelial-to-mesenchymal transformation. These mesenchymal cells further differentiate into the cardiac valves and membranous septa. The cushions themselves function as early valves to control circulation.

We examined TCE’s effects upon valvuloseptal formation using an established *in ovo* chick embryo model (Drake et al. 2006). Exposure was targeted to the period of cushion morphogenesis; studies of cardiomyogenesis will be reported separately (Drake VJ, Koprowski SL, Smith SM, Lough J, unpublished data). Here, we report that TCE exposure at 4 nmol/egg (8 ppb), a dose slightly above the current U.S. EPA MCL for drinking water (5 ppb; U.S. EPA 2004), adversely affects cushion development, cardiac function, and embryo survival. Equimolar exposure to TCA and trichloroethanol (TCOH) had similar consequences.

## Materials and Methods

### Animals

All studies used fertile white leghorn chicken eggs, Babcock strain (University of Wisconsin–Madison) except that the cardiovascular function studies, performed at the University of Utah, used fertile white leghorn eggs, Bovan strain (Utah State University, Logan, UT). We found no differences in the TCE responses of these strains (Drake VJ, Koprowski SL, Smith SM, Lough J, unpublished data). Eggs were incubated at 37.5°C and 70% relative humidity. Embryos were staged using established criteria (Hamburger and Hamilton 1951). Embryos did not develop past day 6.25 and did not experience pain or suffering.

### Materials

TCE, TCOH, or TCA (all from Sigma-Aldrich, St. Louis, MO) was dissolved in prewarmed (50°C) phosphate-buffered saline (PBS) to give a 1 mM stock concentration. These stock solutions were subsequently diluted in prewarmed PBS and their pH adjusted to 7.4. Solutions were prepared fresh immediately before each experiment.

### Embryonic TCE exposure

**Figure 1:**
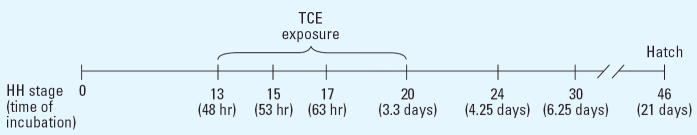
TCE treatment protocol. TCE was administered four times during cardiac cushion formation, at stages HH13, HH15, HH17, and HH20. Embryos were assessed at HH24 or HH30.

We used an established model of *in ovo* teratogen administration in which the teratogen is directly injected into the center of the yolk via a hole at the egg’s blunt end. This method does not cause developmental anomalies in control embryos (Drake et al. 2006). We used a repeated-exposures protocol to simulate the repeated, modest TCE exposures that a human embryo might experience. Embryos received a total of four injections of TCE or carrier solvent PBS at Hamburger and Hamilton (HH) stages HH13, HH15, HH17, and HH20 (2–3.3 days of incubation; Figure 1). These treatment times spanned the major events of cardiac cushion formation, from cushion induction through mesenchyme transformation and migration. We tested three TCE doses that bracket the U.S. EPA MCL: 0.4 ppb, 8 ppb, and 400 ppb (Table 1). Each dose was divided into four 50 μL exposures: 0 nmol/injection (PBS vehicle, 0 nmol total), 0.05 nmol/injection (0.2 nmol total), 1.0 nmol/injection (4 nmol total), and 50 nmol/injection (200 nmol total). Some embryos instead received TCOH or TCA at a dose of 1.0 nmol/injection (4 nmol total). After each injection, the hole was sealed and the eggs reincubated.

### Apoptosis assessment

HH24 embryos were sectioned to consistently visualize the OFT and AVC cushions at the level of the atrial septum and the incipient superior–inferior cushion fusion, respectively. Cushion morphogenesis is largely complete by HH24, and the cushions function as rudimentary valves. Apoptotic cells were detected using a modified terminal deoxynucleotidyl transferase-mediated dUTP nick end-labeling (TUNEL) protocol (Gavrieli et al. 1992); nicked DNA was end-labeled via 5-bromo-2′-deoxyuridine 5′-triphosphate (BrdU; Sigma-Aldrich) incorporation and visualized by immunostaining with anti–BrdU antibody (G3G4; Developmental Studies Hybridoma Bank, Iowa City, IA) and Alexa Fluor 488–conjugated antibody (Molecular Probes, Eugene, OR). All nuclei were visualized by propidium iodide (PI) counterstaining. The percentage of TUNEL-positive nuclei in the OFT and AVC mesenchymal nuclei was determined by treatment-blinded observers. Care was taken to exclude myocytes and endocardiocytes. Two sections per cushion comprising approximately 300 OFT or AVC cells per section were counted.

### Cellularity and proliferation assessment

BrdU (50 μL of 10 mM stock) was directly applied to HH24 embryos *in ovo*; 4 hr later, embryos were fixed, and incorporated BrdU was detected as previously described (Wendler et al. 2003). Sections through the OFT and AVC cushions were prepared as described above for the apoptosis studies. We determined the percentage of proliferating cushion mesenchymal cells for each embryo by counting the number of BrdU-labeled and PI-labeled cells in two sections each through the OFT (> 200 cells/section) and the AVC (> 300 cells/section) cushions. All counts were made at the same position within each heart, at the level of the atrial septum for the OFT and at the level of incipient superior–inferior cushion fusion for the AVC. We excluded myocytes and endocardiocytes. To ascertain the overall cushion cellularity, PI-labeled cells were counted in these two sections and in two additional, adjacent cranial and caudal sections (total of six sections per heart region), with a mean total of approximately 1,300 OFT cells and approximately 1,700 AVC cells per embryo.

### Hemodynamic assessment

Cardiovascular effects of TCE exposure were assessed at HH24 using established methodology (Clark et al. 1986; Hu and Clark 1989). In brief, the egg was positioned blunt end up on a microscope stage. The shell and overlying membranes were removed to expose the embryo. Dorsal aortic and atrioventricular blood velocities were measured with a 20-MHz pulsed-Doppler velocity meter (model 545C-3; Department of Bioengineering, University of Iowa, Iowa City, IA). Dorsal aortic blood velocity was obtained by positioning a 0.75-mm piezoelectric crystal at a 45° angle above the dorsal aorta adjacent to the sinus venosus. Atrioventricular blood velocity was recorded by positioning the crystal at the apex of the ventricle, pointing toward the atrioventricular orifice. Analog waveforms were sampled digitally at 500 Hz via an analog-to-digital board (AT-MIO16; National Instruments, Austin, TX) and viewed with custom analysis software (Labview; National Instruments).

Data were analyzed over five consecutive cardiac cycles for each embryo, and heart rate (beats per min) was determined from the interval of cardiac cycles. The dorsal aortic blood flow (cubic millimeters per second) was calculated by multiplying the integrated velocity curve and the dorsal aortic cross-sectional area; the latter did not differ between PBS- and TCE-exposed groups. Dorsal aortic blood flow includes all blood ejected from the heart except for 10% that is directed cranially (Hu and Clark 1989). The stroke volume index (cubic millimeters per beat) was obtained as the quotient of dorsal aortic blood flow against heart rate. The atrioventricular blood velocity of the diastolic filling has a passive and an active component. The passive phase emerged from end-systole to the onset of the a-wave, and the active phase, from the onset of the a-wave to ventricular pressure upstroke (Hu et al. 1991). Passive atrioventricular blood flow (cubic millimeters per second) was calculated using the equation [passive component area/(passive + active areas)] × dorsal aortic blood flow. Active atrioventricular blood flow (cubic millimeters per second) was calculated using the equation [active component area/(active + passive areas)] × dorsal aortic blood flow.

### Statistical analyses

Values are presented as mean ± SE. Data sets were examined using SigmaStat statistical software (version 2.0; Systat Software Inc., Point Richmond, CA). Normally distributed data were subjected to an unpaired, two-tailed *t*-test employing the appropriate variance parameter (equal or unequal variance). Data not normally distributed were examined using the Mann-Whitney *U*-test. We considered *p* < 0.05 significant.

## Results

### TCE decreased embryo survival

**Figure 2:**
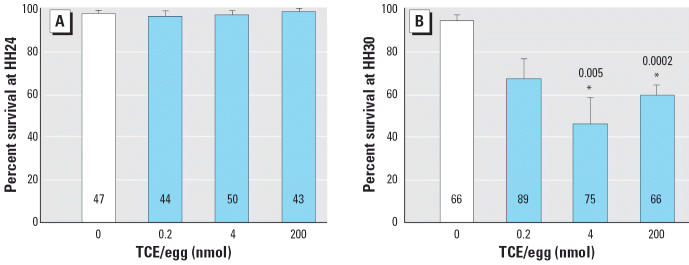
Effects of TCE on chick embryo survival at (*A*) HH24 and (*B*) HH30. Values shown are mean percent survival (± SE) for embryos exposed to TCE during cardiac cushion formation. Each panel represents the mean of five experiments; numbers within bars indicate the total number of embryos. *Significantly different from 0 nmol treatment (*p* < 0.01); *p*-values are given above bars.

Chick embryos were exposed to TCE (total of 0.2, 4, or 200 nmol/egg) during cardiac cushion formation (HH13, HH15, HH17, and HH20). Twenty-two hours after the last injection (HH24; 4.25 days’ incubation), embryo survival, defined as the presence of a beating heart, was normal for all four treatments (Figure 2A). However, at HH30 (6.25 days’ incubation; Figure 2B), there was significantly reduced survival for embryos receiving 4 or 200 nmol TCE/egg. Gross malformations at HH24 and HH30 were rare and did not differ in frequency or appearance between the control and experimental groups.

### Cardiac cushion morphology and cellularity

**Figure 3:**
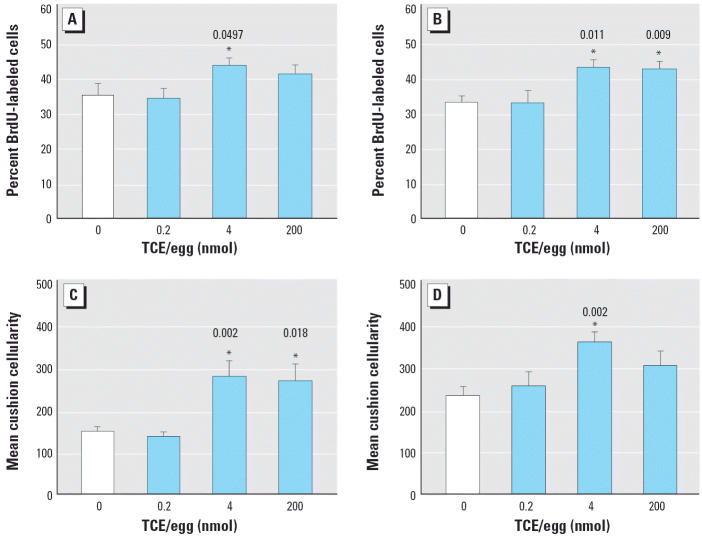
Effects of TCE on cardiac cushion proliferation and cellularity in HH24 chick embryos. Embryos were exposed to TCE during cushion development. Percentage of BrdU-labeled cushion mesenchyme in (*A*) OFT cushions and (*B*) AVC cushions. Total mesenchymal cellularity in the (*C*) OFT cushions and (*D*) AVC cushions. Values shown are mean ± SE. Experiments were performed in triplicate; the total number of embryos evaluated was 8 for 0 nmol, 7 for 0.2 nmol, 10 for 4 nmol, and 9 for 200 nmol. *Significantly different from 0 nmol treatment; *p*-values are given above bars.

TCE-exposed hearts at HH24 were not grossly dysmorphic. Myocardial wall thickness and trabeculation were superficially normal. Because cardiac cushion development may be sensitive to TCE (Boyer et al. 2000), we evaluated the cushions in detail. The cellular morphology of cushion mesenchyme, cardiomyocytes, and endocardiocytes was unaffected by TCE exposure for all concentrations tested (data not shown). TCE did not alter the incidence of apoptosis in the AVC and OFT cushions, except at the 200 nmol dose, which caused a modest but significant increase in the OFT mesenchyme (Table 2). In contrast, TCE exposure at 4 nmol/egg significantly increased the proliferative index in the OFT and AVC cushion mesenchyme (Figure 3A,B). Exposure to 200 nmol TCE/egg also increased proliferation in the AVC cushion (*p* = 0.009) but not in the OFT (*p* = 0.185). The increased cushion mesenchyme proliferation was translated into significant cushion hypercellularity for both the OFT and AVC of TCE-exposed embryos (Figure 3C,D).

### Effects of TCE metabolites

**Figure 4:**
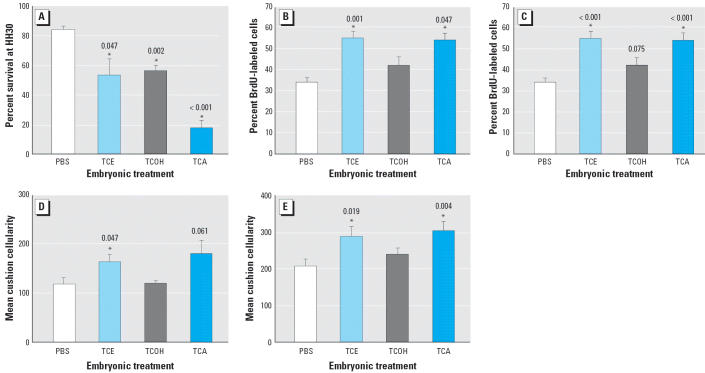
Effects of TCE and its metabolites on embryo survival and cushion development. Embryos were treated with PBS or with 4 nmol/egg of TCE, TCA, or TCOH during cushion development. (*A*) Mean embryo survival ± SE at HH30 (*n* = 3 experiments; values represent 32 embryos for PBS, 46 for TCE, 41 for TCOH, and 34 for TCA). (*B* and *C*) Mean cardiac cushion proliferative index ± SE in OFT (*B*) and AVC (*C*), assessed at HH24 using BrdU incorporation. (*D* and *E*) Mean cushion cellularity of the OFT (*D*) and AVC (*E*). Experiments in *B*–*E* were performed in duplicate and represent 7 embryos for PBS, 7 for TCE, 5 for TCOH, and 7 for TCA. *Significantly different from PBS treatment; *p*-values are given above bars.

TCE metabolites such as TCA are also reported to be cardiac teratogens (Johnson et al. 1998a), and some propose that the proximate teratogen of TCE exposure is TCA (Johnson et al. 1998b). We evaluated the consequences of exposure to the major TCE metabolites TCOH and TCA compared with TCE. All metabolites were provided as sequential 1 nmol doses at HH13, HH15, HH17, and HH20 as described above (4 nmol/egg total). As before (Figure 2B), 4 nmol/egg TCE reduced embryonic survival when assessed at HH30 (Figure 4A). Equimolar exposure to TCOH similarly reduced embryo survival at HH30. An equivalent TCA dose caused the greatest reduction in embryo survival (*p* < 0.001) and was more potent in this aspect than was TCE (*p* = 0.041) or TCOH (*p* = 0.003).

Mesenchymal cell proliferation within the cardiac cushions was assessed in HH24 embryos exposed to these metabolites. Exposure to 4 nmol TCA, but not TCOH, significantly increased cell proliferation in the OFT (Figure 4B) and AVC (Figure 4C) cushions to levels similar to those caused by the parent compound TCE. This increased proliferation was accompanied by increased OFT and AVC cushion hypercellularity (Figure 4D,E).

### TCE exposure alters cardiac hemodynamics

**Figure 5:**
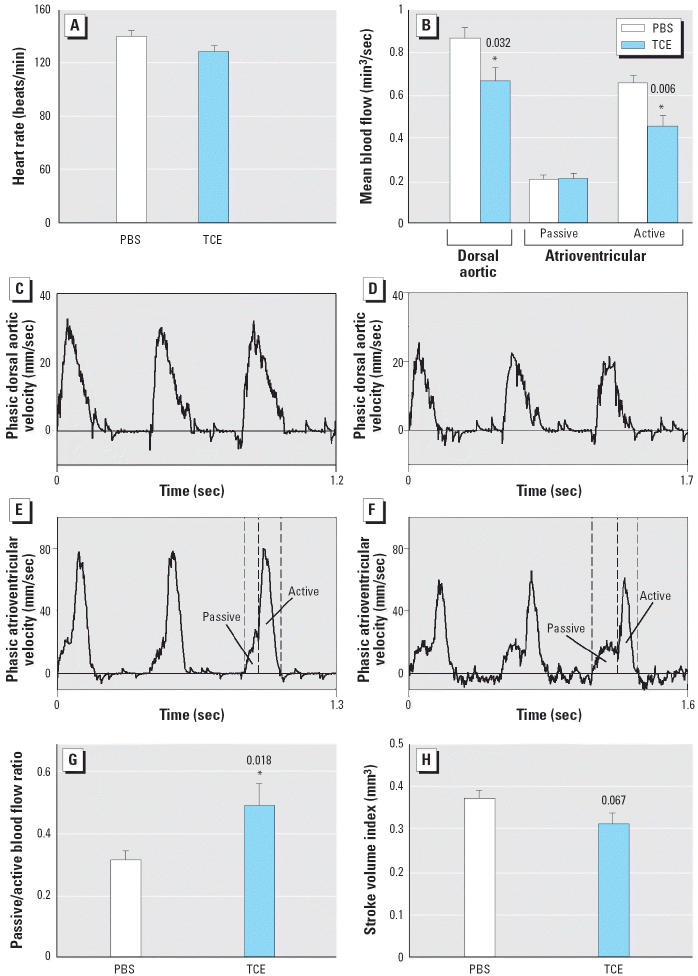
Effects of TCE on cardiovascular function at HH24. Embryos were treated with PBS or 4 nmol TCE during cushion development. Heart rate (*A*) and mean dorsal aortic and atrioventricular (both passive and active components for the atrioventricular) blood flow (*B*) for PBS- and TCE-exposed embryos; values are mean ± SE for 8 PBS or 11 TCE embryos. (*C–F*) Representative dorsal aortic (*C* and *D*) and atrioventricular (*E* and *F*) velocity analog waveforms for a PBS-treated (*C* and *E*) and a TCE-treated (*D* and *F*) embryo; TCE exposure reduced both the dorsal aortic and active atrioventricular velocities. (*G* and *H*) Ratio of passive to active atrioventricular blood flow (*G*) and stroke volume index (*H*) for PBS- and TCE-exposed embryos. *Significantly different from PBS treatment; *p*-values are given above bars.

The cushion hypercellularity and increased mortality caused by 4 nmol/egg TCE prompted an evaluation of cardiac hemodynamics. Pulsed-Doppler assessment of *in ovo* hearts was performed at HH24, when embryo survival was still normal (Figure 2A). The hemodynamic parameters (heart rate, stroke volume, mean blood flow) of PBS-treated embryos were consistent with previously published values for HH24 chick embryos (Broekhuizen et al. 1993; Clark and Hu 1982; Hu and Clark 1989; Hu et al. 1991); hence, the treatment protocol per se did not adversely affect cardiac blood flow. In contrast, TCE exposure altered hemodynamic parameters. Although TCE affected neither cardiac cycle length (474 ± 18 msec vs. 431 ± 13 msec, TCE vs. PBS treatment; *p* = 0.088) nor heart rate (*p* = 0.085; Figure 5A), TCE-treated embryos had a 22.9% reduction in dorsal aortic blood flow (*p* = 0.032; Figure 5B–D). Because TCE did not affect the dorsal aortic diameter (PBS, 0.42 ± 0.008 mm; TCE, 0.42 ± 0.01 mm; *p* = 0.78), this decrease could be attributed to a 30.5% reduction in the active component of atrioventricular blood flow (0.46 ± 0.05 mm^3^/sec vs. 0.66 ± 0.04 mm^3^/sec, TCE vs. PBS treatment; *p* = 0.006; Figure 5B,E,F). The passive-to-active atrioventricular blood flow ratio also was significantly greater in TCE-exposed embryos (*p* = 0.018; Figure 5G). Additionally, TCE treatment was associated with a trend toward a lower stroke volume, although this did not reach statistical significance (*p* = 0.067; Figure 5H). Collectively, these data show that exposure to 4 nmol TCE during cushion morphogenesis reduced the cardiac output of these embryos.

## Discussion

In this article we report that exposure to environmentally relevant TCE doses during cushion formation altered avian heart development. These exposures caused significant reductions in intracardiac blood flow and were associated with increased mortality. These findings provide independent confirmation that TCE exposure alters heart development in a manner that has deleterious, functional consequences. This work does not address the issue of “selectivity” because only the heart was examined. The question of whether TCE is a cardiac teratogen is controversial (Hardin et al. 2004; Johnson et al. 2004). With the exception of increased mortality that was revealed as development progressed (6.25 vs. 4.25 days’ incubation; Figure 2), the cardiac anomalies induced by TCE, although statistically significant, were subtle. Moreover, previous studies of TCE did not evaluate cardiac function, perhaps because of technologic limitations. From these considerations, we conjecture that subtle cellular alterations that nonetheless affect cardiac function have been overlooked until now. Other recent examples of nonobvious embryonic defects that cause remarkable cardiac dysfunction at later stages of development include the disruption of genes encoding the aryl hydrocarbon receptor (Thackaberry et al. 2002) and retinol binding protein (Smith SM, Flentke GR, Lough J, unpublished data; Wendler et al. 2003).

Our results reinforce previous findings that cardiac cushion development is adversely affected by TCE. TCE alters the expression of rat heart genes, some of which regulate epithelial–mesenchymal cell transformation in the cushions (Collier et al. 2003). Substantially higher TCE concentrations than those studied here (parts per million vs. parts per billion) inhibit epithelial detachment and mesenchyme formation in cultured chick AVC explants (Boyer et al. 2000), and this may reflect overt toxicity to these cells.

Importantly, depending on the region affected and its severity, cushion hyperplasia leads to defects ranging from valvular stenosis, cardiac regurgitation, and reduced chamber flow to overt CHDs, including failed atrial or ventricular septation, aortic or pulmonary aortic stenosis, double-outlet right ventricle, and transposition of the great arteries (Chen et al. 2000; Costell et al. 2002; Galvin et al. 2000; Iwamoto et al. 2003; Lakkis and Epstein 1998). These are among the most common CHDs observed in animals exposed to TCE and dichloroethylene, which include stenosis of the atrioventricular, pulmonary, and aortic valves; aortic and pulmonary artery hypoplasia; and disrupted atrial and ventricular septation (Goldberg et al. 1992; Johnson et al. 1998a; Loeber et al. 1988). Although the cause of the cushion hypercellularity seen here is unknown, the increased proliferation without a commensurate rise in apoptosis is likely contributory. TCE might also affect cues that induce the mesenchymal transition of endocardial cells (Boyer et al. 2000; Collier et al. 2003), or other participants in valvuloseptal morphogenesis, including the neural crest or the cardiomyocytes themselves. Curiously, the 4 nmol/egg TCE dose, when applied to the cardiomyogenesis window of chick embryos (HH3+ to HH17), causes myocyte and endocardiocyte hyperproliferation (Drake VJ, Koprowski SL, Smith SM, Lough J, unpublished data). This suggests that the TCE-induced proliferation of cushion mesenchyme may not be an isolated phenomenon.

**Figure 6:**
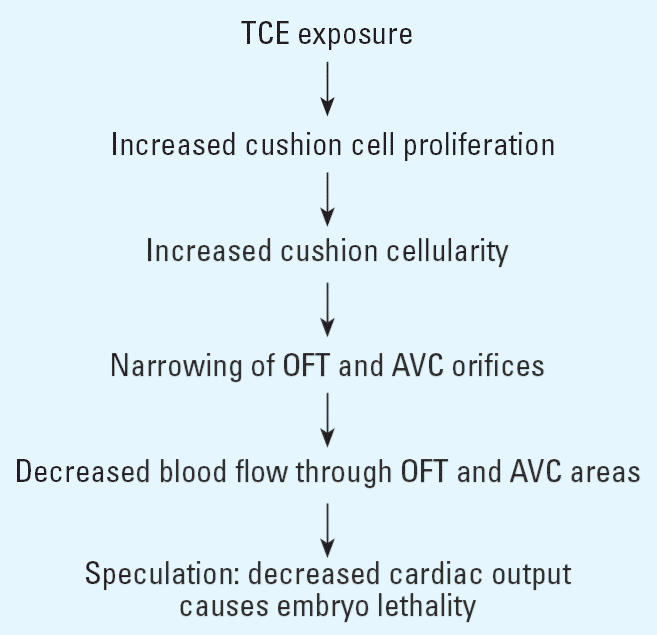
Possible consequences of TCE exposure to cardiac development and function.

The cardiac cushions are critical structures that arise early during heart morphogenesis to form the valves, anchor the atrial and ventricular muscular septa, and divide the OFT into the aorta and pulmonary artery. They also act as rudimentary valves that control chamber filling, prevent backflow, and sustain cardiac output and arterial pressure. Their function is critical to embryonic survival because the embryo’s expanding vascularization in response to rapid growth requires the heart to continuously increase its cardiac output and meet this growing demand (Campbell et al. 1992). Perhaps because the early embryonic heart is morphologically simple, its structure can adjust to small changes in hemodynamic load (Sedmera et al. 1999); in turn, subtle changes in cardiac structure may cause disproportionate hemodynamic responses. Hence, the primary defect caused by TCE could be anatomical or functional. With respect to the former, as outlined in Figure 6, cushion hypercellularity would narrow the OFT and AVC orifices, consequently reducing blood flow during a time of high growth demand, thereby causing mortality. Cardiomyocyte dysfunctions, although not identified, could be contributory. Changes in flow rate and pressure would in turn remodel the growing OFT and AVC septa and valves. Regardless of the initial target, such changes are often lethal.

The TCE doses studied here may have relevance for environmental exposure. Of the three doses tested—0.2 nmol/egg (0.4 ppb), 4 nmol/egg (8 ppb), and 200 nmol/egg (400 ppb)—only the latter, which is a dose encountered at contaminated sites (Goldberg et al. 1990; MDPH 1996), appreciably exceeds the 5 ppb U.S. EPA MCL (U.S. EPA 2004). Our study revealed a clear dose–response threshold: whereas TCE at 0.2 nmol/egg did not affect cushion formation or embryo survival, these measures were adversely affected at the two higher doses. The 4 nmol/egg and 200 nmol/egg doses were largely equipotent, suggesting that the hyperproliferative response of cushion cells reached a maximum at the lower dose. Our 4 nmol/egg dose, administered into the yolk, is in a similar range (0.30–0.75 nmol/egg, applied directly to the embryo) previously shown to increase CHD in chick embryos (Loeber et al. 1988). These doses are lower than those causing CHD in the mammalian studies (0.25, 1.5, and 1,100 ppm; Dawson et al. 1990, 1993; Johnson et al. 1998a, 2003), although direct comparison is difficult because the fetal TCE or TCA content was not assessed.

Metabolic activation of TCE to TCA may be necessary for the compound’s cardiac teratogenicity (Johnson et al. 1998b). Compared with TCE and TCOH, TCA most potently decreased embryonic survival (Figure 4A) and was equipotent with TCE in stimulating cushion mesenchymal cell proliferation. Our results clearly reinforce previous findings that TCE is a cardiac teratogen for chick and extend the cardioteratogenicity of TCA to this species. Importantly, this is the first demonstration that developmental TCE exposure adversely affects the hemodynamic activities of the heart. These effects occurred at exposures only slightly above the U.S. EPA MCL for TCE. A parallel application of technologies that evaluate cardiac function *in utero* may offer insights into the controversy of whether cardiac deficits occur in mammals receiving gestational TCE exposure. To that end, we note that thalidomide variably perturbs rodent development but is a potent teratogen for avian and non-rodent species, including humans (Schardein 2000). Advanced molecular and physiologic tests across several model species should help resolve this question for TCE.

## Figures and Tables

**Table 1:** TCE exposure per egg and the conversion factors.

	Total amount of TCE injected per egg		
Amount per injection (× 4 injections)	ppb/egg	nmol/egg	Concentration (nM)^a^
0.1 ppb or 0.05 nmol	0.4	0.2	3
2 ppb or 1 nmol	8.0	4.0	60
100 ppb or 50 nmol	400	200	3,000

a Assumes a mean egg volume of 66.7 mL.

**Table 2:** Effects of TCE exposure on apoptosis in the cardiac cushions.^a^

	Cushion apoptosis (% TUNEL-positive cells)	
TCE/egg (nmol)	OFT	AVC
0	0.529 ± 0.29	0.326 ± 0.15
0.2	1.721 ± 0.61	0.192 ± 0.19
4	1.211 ± 0.39	0.214 ± 0.10
200	2.130 ± 0.55*	0.392 ± 0.21

Values shown are mean ± SE.

a Experiments were performed in duplicate; *n* = 5–10 embryos per dose.

* *p* < 0.01 versus 0 nmol TCE/egg (Mann-Whitney *U*-test).

## References

[b1-ehp0114-000842] Armstrong SR, Green LC (2004). Chlorinated hydrocarbon solvents. Clin Occup Environ Med.

[b2-ehp0114-000842] Bove FJ, Fulcomer MC, Klotz JB, Esmart J, Dufficy EM, Savrin JE (1995). Public drinking water contamination and birth outcomes. Am J Epidemiol.

[b3-ehp0114-000842] Boyer AS, Finch WT, Runyan RB (2000). Trichloroethylene inhibits development of embryonic heart valve precursors *in vitro*. Toxicol Sci.

[b4-ehp0114-000842] Broekhuizen ML, Mast F, Struijk PC, van der Bie W, Mulder PG, Gittenberger-de Groot AC (1993). Hemodynamic parameters of stage 20 to 35 chick embryo. Pediatr Res.

[b5-ehp0114-000842] Campbell KA, Hu N, Clark EB, Keller BB (1992). Analysis of dynamic atrial dimension and function during early cardiac development in the chick embryo. Ped Res.

[b6-ehp0114-000842] Chen B, Bronson RT, Klaman LD, Hampton TG, Wang J, Green PJ (2000). Mice mutant for Egfr and Shp2 have defective cardiac semilunar valvulogenesis. Nat Genet.

[b7-ehp0114-000842] Clark EB, Hu N (1982). Developmental hemodynamic changes in the chick embryo from stage 18 to 27. Circ Res.

[b8-ehp0114-000842] Clark EB, Hu N, Dummett JL, Vandekieft GK, Olson C, Tomanek R (1986). Ventricular function and morphology in chick embryo from stages 18 to 29. Am J Physiol.

[b9-ehp0114-000842] Collier JM, Selmin O, Johnson PD, Runyan RB (2003). Trichloroethylene effects on gene expression during cardiac development. Birth Defects Res A.

[b10-ehp0114-000842] Costell M, Carmona R, Gustafsson E, Gonzalez-Iriarte M, Fassler R, Munoz-Chapuli R (2002). Hyperplastic conotruncal endocardial cushions and transposition of the great arteries in perlecan-null mice. Circ Res.

[b11-ehp0114-000842] Dawson BV, Johnson PD, Goldberg SJ, Ulreich JB (1990). Cardiac teratogenesis of trichloroethylene and dichloroethylene in a mammalian model. J Am Coll Cardiol.

[b12-ehp0114-000842] Dawson BV, Johnson PD, Goldberg SJ, Ulreich JB (1993). Cardiac teratogenesis of halogenated hydrocarbon-contaminated drinking water. J Am Coll Cardiol.

[b13-ehp0114-000842] Dorfmueller MA, Henne SP, York RG, Bornschein RL, Manson JM (1979). Evaluation of teratogenicity and behavioral toxicity with inhalation exposure of maternal rats to trichloroethylene. Toxicology.

[b14-ehp0114-000842] Drake VJ, Koprowski SL, Lough JW, Smith SM (2006). Gastrulating chick embryo as a model for evaluating teratogenicity: a comparison of three approaches. Birth Defects Res A Clin Mol Teratol.

[b15-ehp0114-000842] Fisher JW, Channel SR, Eggers JS, Johnson PD, MacMahon KL, Goodyear CD (2001). Trichloroethylene, trichloroacetic acid, and dichloroacetic acid: do they affect fetal rat heart development?. Int J Toxicol.

[b16-ehp0114-000842] Galvin KM, Donovan J, Lynch CA, Meyer RI, Paul RJ, Lorenz JN (2000). A role for Smad6 in development and homeostasis of the cardiovascular system. Nat Genet.

[b17-ehp0114-000842] Gavrieli Y, Sherman Y, Ben-Sasson SA (1992). Identification of programmed cell death in situ via specific labeling of nuclear DNA fragmentation. J Cell Biol.

[b18-ehp0114-000842] Goldberg SJ, Dawson BV, Johnson PD, Hoyme HE, Ulreich JB (1992). Cardiac teratogenicity of dichloroethylene in a chick model. Pediatr Res.

[b19-ehp0114-000842] Goldberg SJ, Lebowitz MD, Graver EJ, Hicks S (1990). An association of human congenital cardiac malformations and drinking water contaminants. J Am Coll Cardiol.

[b20-ehp0114-000842] Hamburger V, Hamilton H (1951). A series of normal stages in the development of the chick embryo. J Morphol.

[b21-ehp0114-000842] Hardin BD, Kelman BJ, Brent RL (2004). Trichloroethylene and cardiac malformations. Environ Health Perspect.

[b22-ehp0114-000842] Hu N, Clark EB (1989). Hemodynamics of the stage 12 to stage 29 chick embryo. Circ Res.

[b23-ehp0114-000842] Hu N, Connuck DM, Keller BB, Clark EB (1991). Diastolic filling characteristics in the stage 12 to 27 chick embryo ventricle. Pediatr Res.

[b24-ehp0114-000842] Iwamoto R, Yamazaki S, Asakura M, Takashima S, Hasuwa H, Miyado K (2003). Heparin-binding EGF-like growth factor and ErbB signaling is essential for heart function. Proc Natl Acad Sci USA.

[b25-ehp0114-000842] Johnson PD, Dawson BV, Goldberg SJ (1998a). Cardiac teratogenicity of trichloroethylene metabolites. J Am Coll Cardiol.

[b26-ehp0114-000842] Johnson PD, Dawson BV, Goldberg SJ (1998b). A review: trichloroethylene metabolites: potential cardiac teratogens. Environ Health Perspect.

[b27-ehp0114-000842] Johnson PD, Dawson BV, Goldberg SJ, Mays MZ (2004). Trichloroethylene: Johnson et al.’s response. Environ Health Perspect.

[b28-ehp0114-000842] Johnson PD, Goldberg SJ, Mays MZ, Dawson BV (2003). Threshold of trichloroethylene contamination in maternal drinking waters affecting fetal heart development in the rat. Environ Health Perspect.

[b29-ehp0114-000842] Lakkis MM, Epstein JA (1998). Neurofibromin modulation of ras activity is required for normal endocardial-mesenchymal transformation in the developing heart. Development.

[b30-ehp0114-000842] Loeber CP, Hendrix MJC, Diez de Pinos S, Goldberg SJ (1988). Trichloroethylene: a cardiac teratogen in developing chick embryos. Pediatr Res.

[b31-ehp0114-000842] MDPH. 1996. Final Report of the Woburn Environmental and Birth Study. Cambridge, MA:Massachusetts Department of Public Health, Centers for Disease Control and Prevention, Massachusetts Health Research Institute.

[b32-ehp0114-000842] Miller JW, Uden PC (1983). Characterization on nonvolatile aqueous chlorination products of humic substances. Environ Sci Technol.

[b33-ehp0114-000842] Ou J, Ou Z, McCarver DG, Hines RN, Oldham KT, Ackerman AW (2003). Trichloroethylene decreases heat shock protein 90 interactions with endothelial nitric oxide synthase: implications for endothelial cell proliferation. Toxicol Sci.

[b34-ehp0114-000842] Person AD, Klewer SE, Runyan RB (2005). Cell biology of cardiac cushion development. Int Rev Cytol.

[b35-ehp0114-000842] Schardein JL 2000. Thalidomide: the prototype teratogen. In: Chemically Induced Birth Defects. 3rd ed. New York:Marcel Dekker, 89–119.

[b36-ehp0114-000842] Schwetz BA, Leong BKJ, Gehring PJ (1975). The effect of maternally inhaled trichloroethylene, perchloroethylene, methyl chloroform, and methylene chloride on embryonal and fetal development in mice and rats. Toxicol Appl Pharmacol.

[b37-ehp0114-000842] Sedmera D, Pexieder T, Rychterova V, Hu N, Clark EB (1999). Remodeling of chick embryonic ventricular myoarchitecture under experimentally changed loading conditions. Anat Rec.

[b38-ehp0114-000842] Selmin O, Thorne PA, Caldwell PT, Johnson PD, Runyan RB (2005). Effects of trichloroethylene and its metabolite trichloroacetic acid on the expression of vimentin in the rat H9c2 cell line. Cell Biol Toxicol.

[b39-ehp0114-000842] Shaw GM, Schulman J, Frisch JD, Cummins SK, Harris JA (1992). Congenital malformations and birthweight in areas with potential environmental contamination. Arch Environ Health.

[b40-ehp0114-000842] Shaw GM, Swan SH, Harris JA, Malcoe LH (1990). Maternal water consumption during pregnancy and congenital cardiac anomalies. Epidemiology.

[b41-ehp0114-000842] Thackaberry EA, Gabaldon DM, Walker MK, Smith SM (2002). Aryl hydrocarbon receptor null mice develop cardiac hypertrophy and increased hypoxia-inducible factor 1alpha in the absence of cardiac hypoxia. Cardiovasc Toxicol.

[b42-ehp0114-000842] U.S. EPA. 1997. Community Water System Survey. Vol 1. Overview. EPA 815-R-97-001A. Washington, DC:U.S. Environmental Protection Agency.

[b43-ehp0114-000842] U.S. EPA. 2004. 2004 Edition of the Drinking Water Standards and Health Advisories. EPA 822-R-04-005. Office of Water. Washington, DC:U.S. Environmental Protection Agency.

[b44-ehp0114-000842] Wallace LA, Pellizzari ED, Hartwell TD, Sparacino C, Whitmore R, Sheldon L (1987). The TEAM study: personal exposures to toxic substances in air, drinking water, and breath of 400 residents of New Jersey, North Carolina, and North Dakota. Environ Res.

[b45-ehp0114-000842] Waters EM, Gerstner HB, Huff JE (1977). Trichloroethylene. I. An overview. J Toxicol Environ Health.

[b46-ehp0114-000842] Wendler CC, Schmoldt A, Flentke GR, Case LC, Quadro L, Blaner WS (2003). Increased fibronectin deposition in embryonic hearts of retinol-binding protein-null mice. Circ Res.

[b47-ehp0114-000842] Wu C, Schaum J (2000). Exposure assessment of trichloroethylene. Environ Health Perspect.

[b48-ehp0114-000842] Yauck JS, Malloy ME, Blair K, Simpson PM, McCarver DG (2004). Proximity of residence to trichloroethylene-emitting sites and increased risk of offspring congenital heart defects among older women. Birth Defects Res A.

